# Financial Outcomes of “Bagging” Oncology Drugs Among Privately Insured Patients With Cancer

**DOI:** 10.1001/jamanetworkopen.2023.32643

**Published:** 2023-09-07

**Authors:** Ya-Chen Tina Shih, Ying Xu, James Ching Yao

**Affiliations:** 1 UCLA Jonsson Comprehensive Cancer Center, Department of Radiation Oncology, David Geffen School of Medicine, University of California, Los Angeles; 2 Department of Health Services Research, The University of Texas MD Anderson Cancer Center, Houston; 3 Department of Gastrointestinal Medical Oncology, The University of Texas MD Anderson Cancer Center, Houston

## Abstract

This cohort study examines the difference in per-patient per-month costs between drugs distributed under “bagging,” a drug delivery model that requires patients to obtain physician-administered medications via pharmacies, and traditional buy-and-bill practice.

## Introduction

Traditionally, medications that must be delivered via intravenous infusion have been purchased by physicians and administered directly to patients in medical settings (ie, buy and bill); insurers covered them under medical benefit. Recently, some insurers have started “bagging”—a drug delivery model that requires patients to obtain physician-administered medications via pharmacies, often specialty pharmacies, and either have the medications shipped to physicians’ offices or to patients who then bring the medications to their physician’s office to be administered.^1^ Medications distributed under bagging practice are covered by pharmacy benefit. Patient safety concerns have prompted the American Hospital Association^2^ and the American Society of Clinical Oncology^3^ to issue position statements recommending that policymakers restrict bagging practice. As of 2021, 3 states (Arkansas, Louisiana, and Virginia) have enacted laws prohibiting insurers from mandating bagging practice.^4^ Despite concerns voiced by professional associations and growing legislative efforts to restrict bagging practice, there is limited information on the prevalence of bagging in oncology and its financial effect. This cohort study fills an important knowledge gap.

## Methods

We selected 50 cancer drugs with the highest total spending from the 2020 Medicare Part B drug spending dashboard.^5^ Using 2019 to 2020 data from the MarketScan Commercial Claims and Encounter Database, we identified these drugs from medical and pharmacy claims via their corresponding current procedural terminology and national drug codes, respectively, among patients with cancer (eFigure in Supplement 1). We defined bagging as having physician-administered drugs billed to pharmacy benefit and classified these drugs as immunotherapy or targeted therapy, supportive care drugs, and all other anticancer agents. We calculated per-patient per-month (PPPM) insurance payment and out-of-pocket (OOP) expense separately from medical and pharmacy claims and conducted regression analyses to examine the difference in mean and median PPPM costs between drugs distributed under bagging and buy-and-bill practice. The institutional review board at The University of Texas MD Anderson Cancer Center deemed this study exempt from review owing to use of deidentified data. The study followed the STROBE reporting guideline for cohort studies.

## Results

**Figure 1.**
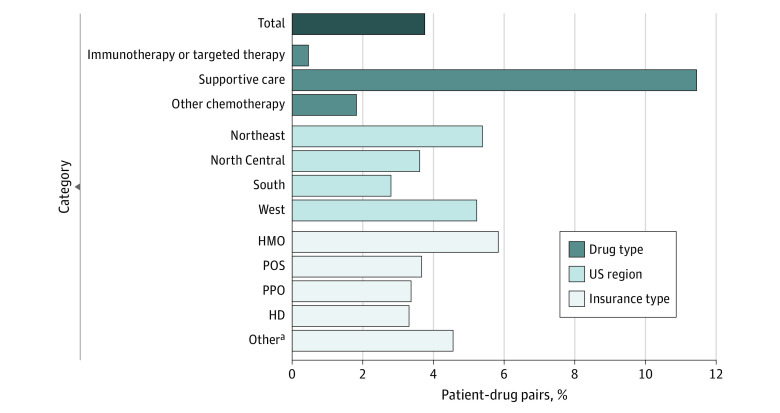
Prevalence of “Bagging” Practice HD indicates high deductible; HMO, health maintenance organization; POS, point of service; PPO, preferred provider organization. ^a^The Other category includes comprehensive plans, exclusive provider organization plans, basic/major medical plans, and capitated or partially capitated POS plans.

**Figure 2.**
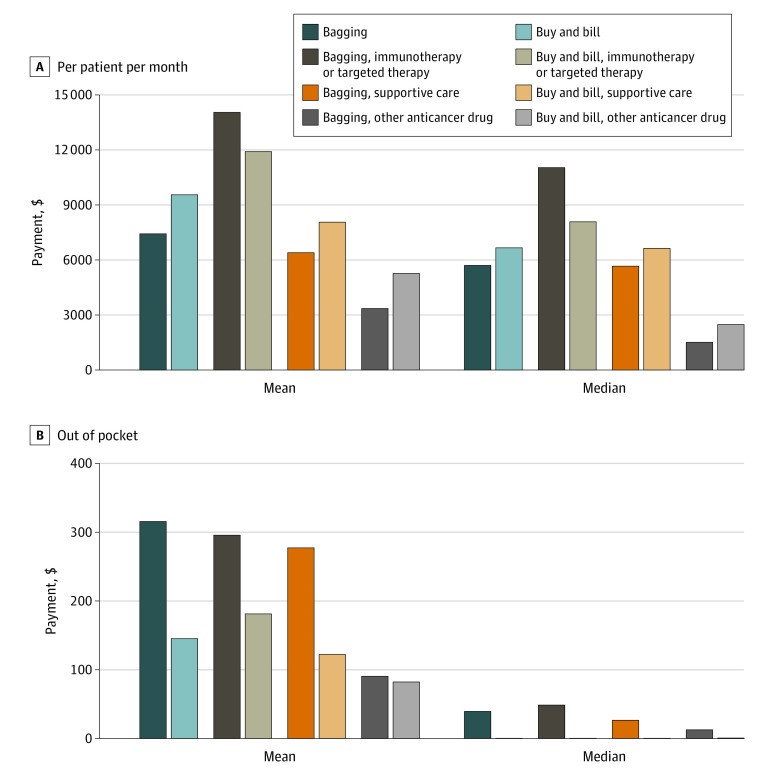
Per-Patient Per-Month and Out-of-Pocket Payments for Drugs Distributed Under “Bagging” vs Buy-and-Bill Practice Payment was obtained from claims associated with specific anticancer drugs and did not include costs of drug administrations. Out-of-pocket payments were the sum of co-payments, coinsurance, and deductibles associated with specific drug claims. The per-member per-month amount was calculated by summing paid or out-of-pocket payments associated with claims for anticancer drugs divided by months of exposure (ie, total number of months a patient with cancer was treated with any anticancer drugs). Means associated with bagging vs buy-and-bill practice were adjusted means derived from generalized linear regression model (Gamma family, log link) that controlled for patients’ age, sex, US region, insurance plan type, and drug category. Medians were adjusted medians derived from quantile regression that controlled for patients’ age, sex, US region, insurance plan type, and drug category.

The study cohort included 113 076 patient-drug pairs (mean patient age, 55.9 years; 68.4% female). Among the patient-drug pairs, 53.1%, 27.6%, and 19.3% were immunotherapy or targeted therapy, supportive care, and other anticancer agents, respectively. Bagging accounted for 3.7% of patient-drug pairs, ranging from less than 1% of immunotherapy or targeted therapy to 11.4% of supportive care products. Variations were also observed by US region and plan type (Figure 1). Adjusted mean insurance payments PPPM were statistically significantly lower for drugs distributed under bagging vs buy and bill ($7405 [95% CI, $7111-$7700] vs $9547 [95% CI, $9471-$9622]; *P* < .001); the same pattern was observed in adjusted median payment ($5746 [95% CI, $5448-$6043] vs $6681 [95% CI, $6624-$6737]) (Figure 2A). Adjusted mean and median OOP payment PPPM was higher for bagging practice vs buy and bill (mean: $315 [95% CI, $278-$351] vs $145 [95% CI, $141-$148]; median: $93.60 [95% CI, $39.50-$39.70] vs $0 [95% CI, $0-$0]; Figure 2B). Stratified analyses showed these patterns were primarily associated with supportive care drugs.

## Discussion

Bagging practice was more common for intravenous support care drugs and infrequent for immunotherapy or targeted therapy. Proponents of bagging practice have emphasized its economic benefit to payers and patients, including the potential of lower premium from overall cost reduction.^6^ However, bagging in oncology was associated with lower payment for insurers (except for immunotherapy or targeted therapy) but higher OOP payment for patients, suggesting payers and patients do not equally benefit from bagging practice financially. Bagging can present challenges for physicians. For example, electronic health record builds of treatment plans typically include supportive medications preferred by and on formulary at the infusion facility. If bagging introduces an alternative supportive medication, modification of treatment plans may or may not be possible depending on what has been built in the site’s electronic health record. Under bagging practice, physician-administered drugs can be shipped to physicians’ offices (known as *white bagging*) or to patients’ residences, then patients bring the drugs to their physician’s office (know as *brown bagging*). A limitation of this analysis was that we were not able to differentiate between brown and white bagging in claims data. As more states are considering legislations to restrict bagging practice,^4^ much research is needed to better understand factors driving the diffusion of bagging in oncology and to evaluate how this practice affects clinical outcomes.

## Notes

Supplement 1. **eFigure.** Ascertainment of Study Cohort

Supplement 2.
Data Sharing Statement

## References

[1] Catizone CA. White and Brown Bagging Emerging Practices, Emerging Regulation. National Association of Boards of Pharmacy; 2018.

[2] Health insurer specialty pharmacy policies threaten patient quality of care. American Hospital Association. March 2021. Accessed August 8, 2023. https://www.aha.org/system/files/media/file/2021/03/AOMarch8white-bagging-0221.pdf

[3] “Brown bagging” and “white bagging” of chemotherapy drugs. American Society of Clinical Oncology. Accessed August 8, 2023. https://old-prod.asco.org/sites/new-www.asco.org/files/content-files/advocacy-and-policy/documents/2021-White-Brown-Bagging-Update.pdf

[4] White-bagging legislation gains popularity in state legislatures. Avalere. April 25, 2022. Accessed August 8, 2023. https://avalere.com/insights/white-bagging-legislation-gains-popularity-in-state-legislatures

[5] Medicare Part B spending by drug. Centers for Medicare & Medicaid Services. Accessed August 8, 2023. https://data.cms.gov/summary-statistics-on-use-and-payments/medicare-medicaid-spending-by-drug/medicare-part-b-spending-by-drug

[6] The Pharmacy Benefit Brief: November 2021. PCMA. November 18, 2021. Accessed August 8, 2023. https://www.pcmanet.org/the-pharmacy-benefit-brief-november-2021/

