# Supplementary material for: Financial Outcomes of “Bagging” Oncology Drugs Among Privately Insured Patients With Cancer
Source: JAMA Netw Open. 2023 Sep 7;6(9):e2332643. doi: 10.1001/jamanetworkopen.2023.32643 (PMC10485724; doi:10.1001/jamanetworkopen.2023.32643)
Supplement: Supplement 1. — eFigure. Ascertainment of Study Cohort [file jamanetwopen-e2332643-s001.pdf]

Supplemental Online Content

Shih YCT, Xu Y, Yao JC. Financial outcomes of “bagging” oncology drugs among privately insured patients with cancer. *JAMA Netw Open*. 2023;6(9):e2332643. doi:10.1001/jamanetworkopen.2023.32643

eFigure. Ascertainment of Study Cohort

This supplemental material has been provided by the authors to give readers additional information about their work.

© 2023 Shih YCT et al. *JAMA Network Open.*

eFigure. Ascertainment of Study Cohort

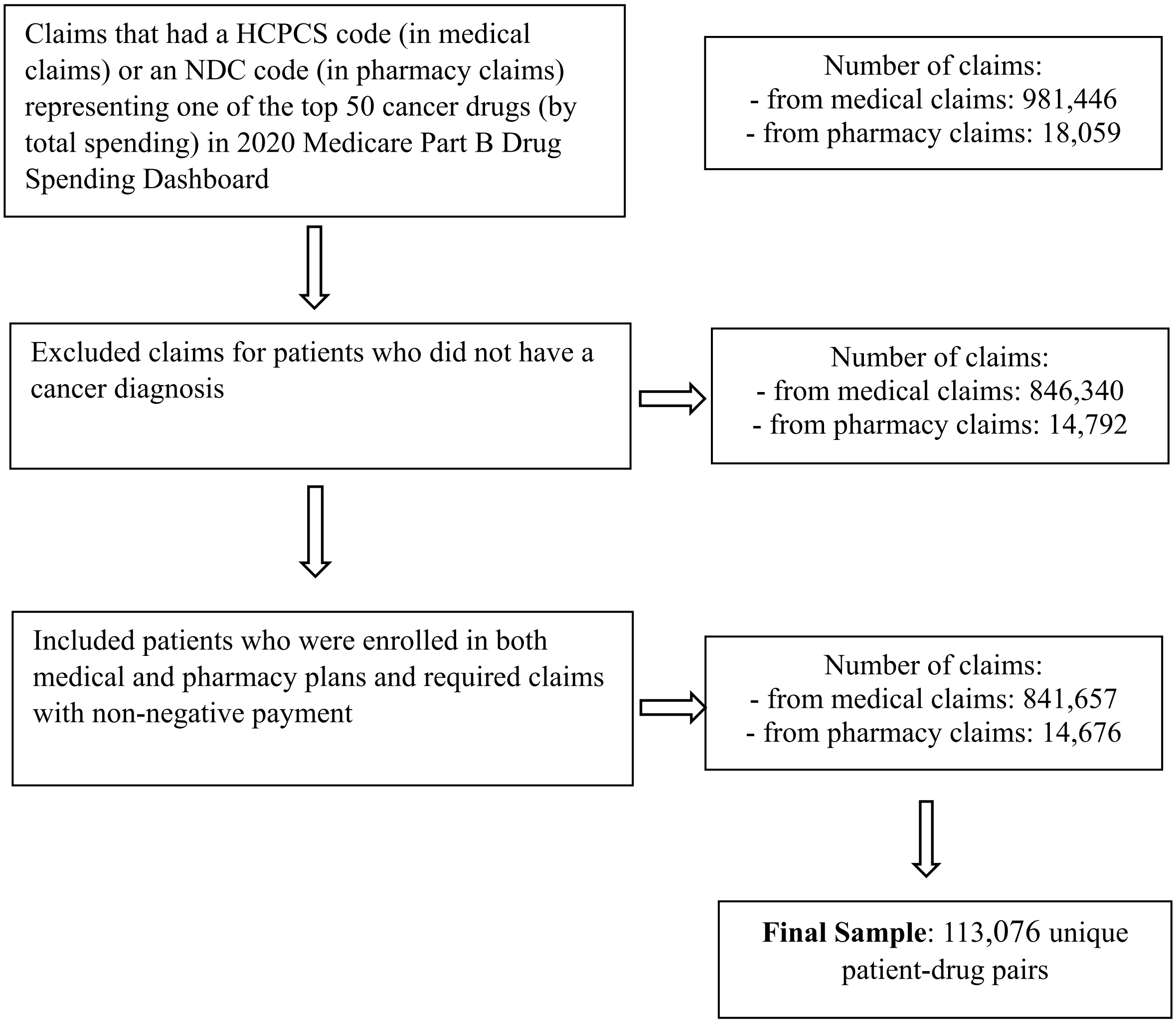

© 2023 Shih YCT et al. *JAMA Network Open.*
