# Supplementary material for: Financial Outcomes of “Bagging” Oncology Drugs Among Privately Insured Patients With Cancer
Source: JAMA Netw Open. 2023 Sep 7;6(9):e2332643. doi: 10.1001/jamanetworkopen.2023.32643 (PMC10485724; doi:10.1001/jamanetworkopen.2023.32643)
Supplement: Supplement 2. — Data Sharing Statement [file jamanetwopen-e2332643-s002.pdf]

Data Sharing Statement

Shih. Financial Outcomes of “Bagging” Oncology Drugs Among Privately Insured Patients With Cancer. *JAMA Netw Open*. Published September 07, 2023.
doi:10.1001/jamanetworkopen.2023.32643

Data
Data available: No

Additional Information
Explanation for why data not available: The raw/processed data required to reproduce the study findings cannot be shared under the data use agreement between University of Texas MD Anderson Cancer Center and Merative, vendor of the MarketScan Research databases.
